# Presence of the Aphid, *Chaetosiphon fragaefolii*, on Strawberry in Argentina

**DOI:** 10.1673/031.010.0901

**Published:** 2010-03-02

**Authors:** Claudia Cédola, Nancy Grecob

**Affiliations:** Centro de Estudios Parasitológicos y de Vectores (CEPAVE- CCT-La Plata-CONICET-UNLP), Calle 2 N° 584 1900 La Plata, Argentina

**Keywords:** *Fragaria × ananassa*, agricultural management, strawberry aphids, aphid complex

## Abstract

Seasonal abundance of the strawberry aphid complex under different agronomic practices in the outskirts of La Plata, Argentina was studied on strawberry, *Fragaria x ananassa* Duchesne (Rosales: Rosaceae). Aphid densities were low in strawberry fields in which insecticides and fungicides were used. In addition to *Aphis gossypii*, *Aphis fabae*, *Mysus persicae* and *Macrosiphum euphorbiae*, the aphid, *Chaetosiphon fragaefolii* (Cockerell) (Homoptera: Aphididae), was recorded for the first time in this horticultural area. Life history and some demographic parameters were calculated for *C. fragaefolii*. The mean duration of nymphal stages was 10.44 days, the oviposition period was 11.8 days, and the mean number of nymph/female/day was 2.4 ± 0.3. Demographic parameters analyzed included the net reproductive rate R_o_ = 14.55 ± 0.096 nymph/female, generation time T=16.91 ± 0.035 days, and the intrinsic rate of increase r_m_ = 0.158 ± (0.004). No parasites were found associated with *C. fragaefolli*. The pathogenic fungus, *Entomophthora planchoniana* Cornu (Zygomycetes: Entomophthorales) was the main mortality factor. Although aphids are not the main pests in strawberry fields, *C. fragaefolii* can be a serious problem because it can transmit several virus diseases of strawberry. Greater knowledge of life history traits and mortality factors of this species is needed in order to design appropriate control strategies.

## Introduction

Herbivores can have both direct and indirect negative impacts on all aspects of plant growth, reproduction and productivity (Whitham et al. 1991). Aphids can reduce crop yield through direct consumption, aesthetic damage and virus transmission.

In agricultural systems, the relationships between different trophic levels are disturbed by the effect of agricultural practices such as the use of insecticides and fertilizers that diminish the diversity and structural complexity of the agroecosystem (Moreau et al. 2006). Thus, the structure of herbivore communities and their natural enemies within the same crop may differ according to different agronomic practices. Bottom up and top down forces drive population dynamics (Walker and Jones 2001). In the first case, for example it has been documented that plant nutritional quality influences insect hervivores (Perrenoud 1990) and many of them such phloem feeding aphids and others, are sensitive to changes in macronutrients such as nitrogen or potassium. Myers and Gratton (2006) found that potassium availability in soil and leaves may play an important role in aphid population dynamics. On the other hand Costamagna and Landis (2006) found that predators exert top down control on aphid dynamics in soybeans crops.

The strawberry, *Fragaria x ananassa* Duchesne (Rosales: Rosaceae) is one of the main crops produced in Argentina for export (Molina et al. 2007). Among the herbivore arthropods that feed on strawberry there are several species of aphids such as *Aphis gossypii* Glover, *Aphis fabae* Scopoli, *Mysus persicae* (Sulzer), *Macrosiphum euphorbiae* Thomas and *Chaetosiphon fragaefolii* Cockerell (Homoptera: Aphididae), (Rondon et al. 2005). Although aphids are not the main pests on strawberries, they can cause serious problems. The strawberry aphid, *C. fragaefolii*, can affect yields because it transmits viruses such as the *strawberry mild yellow edge virus*, *strawberry crinkle virus* and *strawberry mottle virus* (Krczal 1982). In Argentina, this aphid has been cited for strawberry crops in the provinces of Buenos Aires and Córdoba (Cordo et al. 2004) and it is known to be a vector for the *strawberry mottle virus* (Nome and Yossen 1980). In Argentina, the most frequently used method for pest control involves the regular use of insecticides on a calendar basis. However, many strawberry growers are beginning to reduce the use of pesticides and a few producers use no chemicals at all, either due to their cost or in order to obtain premium price for pesticide-free strawberries. As a consequence, different agronomic practices are currently used in strawberry fields.

The goals of the present study were: (1) to record the seasonal abundance of the aphid complex in strawberries under different input of fungicides and pesticides, (2) to obtain information about the life history of *C. fragaefolii* and (3) to estimate its demographic parameters.

## MATERIALS AND METHODS

### Field sampling

Aphid populations were monitored on commercial strawberry fields in Los Hornos and Colonia Urquiza, two localities near La Plata city (province of Buenos Aires) from April to December 2006. Strawberry cultivars used by farmers were Festival, Camarosa and Aromas. Crops under three different agrochemical treatments were surveyed twice a month. Fertilization was similar in all fields, N, P, K in agronomic standard doses for the region: N = 1Kg/ha, P = 0.5 kg /ha and K = 1.7 Kg/ha.. Eleven strawberry crops located in different fields were grouped according to different fungicide and insecticide input.

Four crops of 5000 m^2^ (0.9 m rows by 50 m) fields received a high input of chemicals (HIC) under calendar spray weekly to prevent mites, thrips and aphids. Insecticides used included: New Mectin abamectin at 0.6 lt/ha (AgriMarketing www.agrimarketing.com.ar), methomyl at 250 g/ha (Lannate, Dupont, www.dupont.com), methamidophos at 1.5 lt/ha, (Metamidofos 60 SINER S.A, www.asaprove.com.ar), and dicofol + tetradifon at 1–2 lt/ha (Tetranyl, Triavet S.A, www.triavet.com.ar). The fungicide used was benomyl at 50 gr/ha (Benosem 50 PM, Sembrado S.A, www.asaprove.com.ar).

In five other 10000 m^2^ (0.90 m rows by 50 m) fields, only fungicides (OF) were used to control fungi and earthworms: benomyl at 50 gr/ha (Benosem 50 PM, Sembrado S.A, www.asaprove.com.ar).

In two 5000 m^2^ (0.90 m by 50 m) fields, little insecticide (LI) was used. Abamectin at 0.6 lt/ha (New Mectin) was used once to diminish aphid density.

A total of 237 aphid samples were collected. Systematic sampling was performed by collecting one leaflet at 5–10 m intervals in each row (approximately 100 leaflets). Leaflets, 6 and 7 cm in diameter, were selected at random from mature leaves in the middle level of the plant. The number of leaflets per sample ranged between 90 and 115, depending on the crop size. Individuals of all the aphid species found were counted under stero-microscope and the number of mummies/leaflet was recorded.

When the abundance of *C. fragaefolii* began to increase the samples were placed in Petri dishes lined with filter paper moistened in distilled water and maintained at 25°C, 70% RH with 14:10 L:D to search for signs of natural enemies. Samples were observed every four days to record the development of any fungi or parasitoids that could be present. Infected aphids were preserved in 70% ethanol for further identification.

The effect of different crop management practices on aphid abundance was assessed by profile analysis following von Ende (2001). The first three sample dates were excluded from the analysis due to the number of samples in which no aphids were present. Because not all the samples from each field were collected on the same dates, they were pooled by 4-week periods.

### Life history traits and demographic parameters

The *C. fragaefolii* colony used in the experiments was started using specimens from Colonia Urquiza. They were maintained on strawberry plants under controlled conditions at 21°C, 60–70 RH and 14:10 L:D. Thirty-six viviparous females of *C. fragaefolii* were randomly selected from the colony and individually transferred to the abaxial side of a strawberry leaflet from Centro de Estudios Parasitológicos y de Vectores stock plants. Plants of Festival, Camarosa and Aromas cultivars were transplanted to 2 liter plastic pots with soil plus humus (50:50) outdoors under natural seasonal photoperiod at CEPAVE. The plants grew vigorously without fertilizers or insecticides being used. Each leaflet was placed in a Petri dish. The petioles were covered with moistened cotton and the leaflets were replaced every four days with a fresh mature leaflet. After 24 hours, the female and all of her offspring were removed except for one recently born nymph. These thirty-six nymphs formed a cohort. Nymphal development was observed once a day, and the presence of exuviae was used to determine molting. When the aphids reached the adult stage, the number of progeny produced per adult and adult mortality were recorded daily. Reproductive and post-reproductive periods and longevity were recorded. The following demographic parameters were calculated: net reproductive rate (R_0_), intrinsic rate of increase (r_m_, = ln R_0_ / T), reproductive value (V_x_), and generation time (T) (Begon et al. 1986). The mean and standard error of R_0_, r_m_ and T were estimated using the jackknife method (Krebs 1999; Caswell 1989). Estimations were done using Microsoft Excel.

## RESULTS AND DISCUSSION

Aphid abundance was significantly affected by agricultural management practices (Wilks^1^ Lambda: 0.000001, Rao's R: 524.80, df1: 12, df2: 6, p < 0.000001).

**Figure 1:**
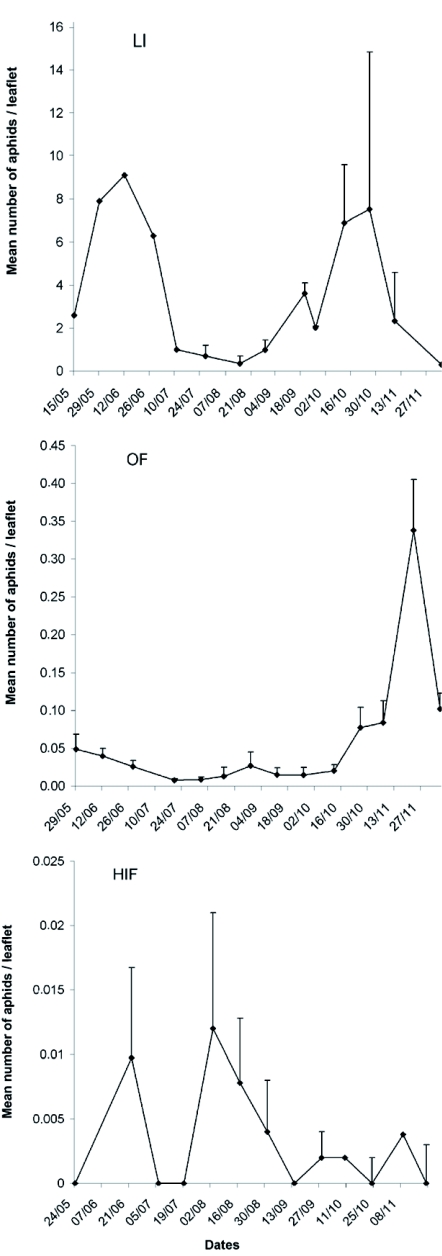
Fluctuation of mean number of *Chaetosiphon fragaefolii* aphids/leaflet in strawberry crops under different agronomic practices (A) LI= little insecticide, (B) OF= only fungicides, (C) HIC= insecticides and fungicides. High quality figures are available online.

In LI the strawberry aphid, *C. fragaefolii*, was the dominant species. Two peaks in population were observed, the first in autumn and the second in spring, and population density gradually decreased towards the end of the sampling period (Figure 1a). Considering the average density throughout the whole sampling period, the mean number of aphids per leaflet was 1.34 ± 1.80 and the maximum density was of 14.84 ± 2.12 aphids /leaflet at the end of October. It should be noted that these estimates are relative densities, as aphids were sampled only from mature strawberry leaves.

**Table 1:**
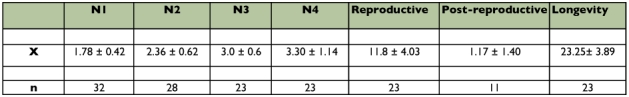
Duration (days) of nymphal stages (N), reproductive, post-reproductive periods, and longevity of ***Ch. fragaefolli*** on strawberry.

*C. fragaefolii* usually infects young leaves. Rondon et al 2005 found that nymphs of *C. fragaefolii* were more frequently found on leaves than on the developing buds but apterous adults predominated on the buds. In most plant species young leaves are more heavily attacked by insects than mature leaves (Slansky and Rodriguez 1987). One reason may be because they are less tough and easier to suck, chew and digest. Another factor that may contribute to high herbivory on young leaves is their greater nutritional value. Young leaves typically have to two to four times the nitrogen content of mature leaves (Mattson and Scriber 1987, Coley and Aide 1991). Diet with higher nitrogen can increase herbivore fitness (Mattson and Scriber 1987). A diet with higher nitrogen can increase herbivore fitness (Mattson and Scriber 1987).

Between July and August aphid densities were very low. The insecticide application to LI crops was made in October, so these low densities could be due to conditions of the temperate climate with a frost period ranging from mid-May until mid-September.

During the spring, *C. fragaefolii* coexisted only with *A. gossypii* in LI, although at very low densities, never being over 6% of the total aphids per sampling date. During September and October the fungus *Entomophthora planchoniana* Cornu (Zygomycetes: Entomophthorales) was found to be present. In samples that came from the field, the mortality due to fungi was between 10.3 % in mid September and 4.5 % in late October when no fungicides were applied. Application of fungicides is known to reduce entomopathogeniic fungi (Lagnaoui and Radcliffe 1998, Kouassi et al. 2003)

*A. gossypii* was parasitized by *Aphidius* sp. and *Praon* sp., in percentages that fluctuated between 18% (20/9/06) and 10% (26/10/06) in all three field types (HIC, OF and LI), but no parasitism was observed on *C. fragaefolii.* This finding coincides with the report of Rondon and Cantliffe (2004) who suggested that the capitate hair constitutes a barrier that prevents parasitism. The ladybird beetle, *Cycloneda sanguinea*, and immature syrphids were the most conspicuous predators observed in these crops.

Figures 1b and 1c show the abundance of all aphids in OF and HIC, respectively. In HIC and OF, *A. gossypii*, *M. euphorbiae* and *M. persicae* were present, but *C. fragaefolii* was not recorded. Agricultural practices did not significantly affect aphid density (Wilks' Lambda: 0.034, Rao's R: 9.31, df_1_: 6, df_2_: 2, p < 0.1). Maximum density in OF was 0.35 aphids /leaflet (late November), and mummies of parasitic wasps, *Aphidius* sp and *Praon* sp. were found in 28.4 % of samples. Predators were scarce in these fields, but *Crysopa lanata* (Neuroptera: Chrysopidae) and immature stages of syrphids were observed but not quantified.

In HIC aphid density was lower than in OF and mummies were observed only twice. Maximum density was 0.012 aphids /leaflet (late August). No predators were observed in these crops.

### Life traits and demographic parameters

The developmental times of immature and adult stages of *C. fragaefolii* are summarized in Table 1. Reproduction began 24 to 36 h after the last molt. Twenty-three out of 32 nymphs reached adulthood after completing four nymphal instars. However, 12.5 % of the total aphids went through a fifth instar that lasted 2.25 ± 0.95 days. Diaz and Ferere (2005) and La Rossa et al. (2000) also reported an extra nymphal instar for the lettuce aphid, *Nasonovia ribisnigri*. The Russian wheat aphid, *Diuraphis noxia*, also exhibited a variable number of instars (four to six) during the course of its development at different temperatures (Michels and Behle 1988; Nowierski et al. 1995).

**Figure 2:**
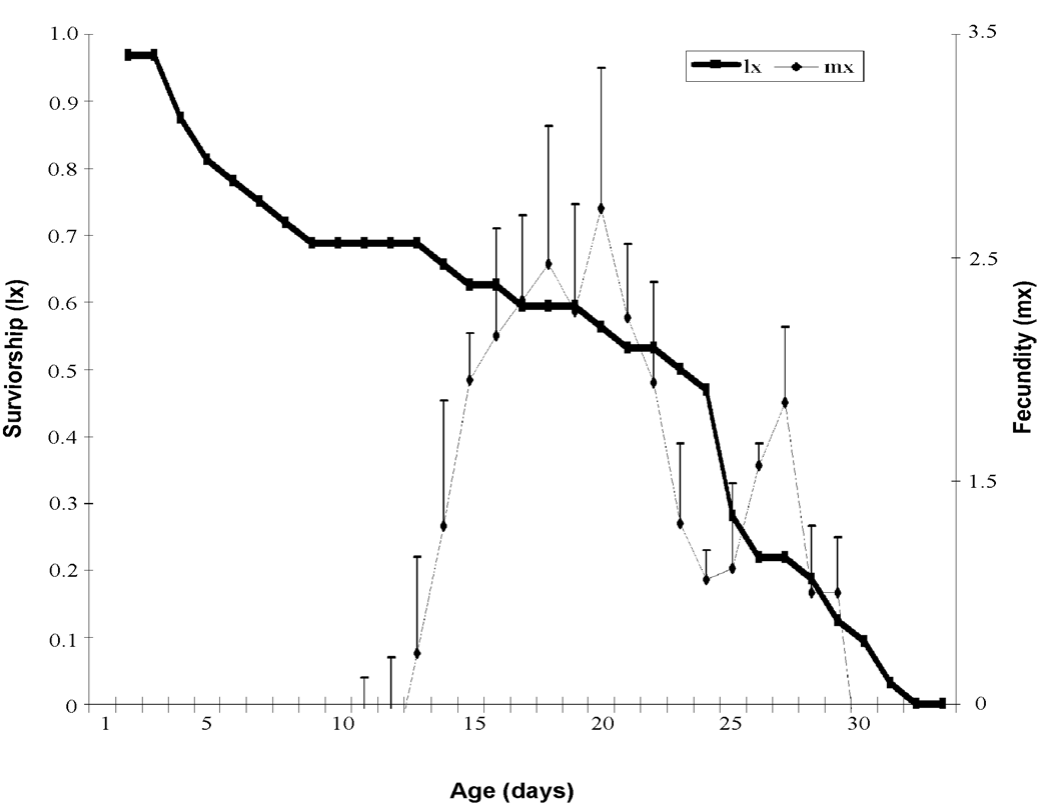
Survival (l_x_) and fecundity (m_x_) of *Chaetosiphon fragaefolii* on strawberry. High quality figures are available online.

The reproductive period represented 51 % of the entire life cycle, leading to stage overlap. Survival (Figure 2) recorded at 11 and 20 days was 31.5 and 8.2 days, respectively, which was higher than the values recorded by Krczal (1982). This parameter decreased slowly and remained relatively constant (l_x_ = 0.63) during the oviposition period (Figure 2). After that it decreased from day 23 until death of all the individuals. The mean number of nymphs/ female/day was 2.4 ± 0.3.

Strawberry cultivar type influences growth capacity of *C. fragaefolii*. For example, females produced an average of 3.7 ± 0.8 nymphs on Festival, 4.3 ± 1.8 nymphs on Carmine and 2.1 ± 1.1 nymphs on Diamond (Rondon and Cantliffe 2004). This information is interesting because it suggests that cultivars could be selected to diminish the growth potential of aphids.

Demographic parameters of *C. fragaefolii* were as follows: the net reproductive rate Ro= 14.55 ± 0.096 nymph/female, generation time T= 16.91 ± 0.035 days and the intrinsic rate of increase r_m_= 0.158 ± (0.004) nymph/female/day. Although the r_m_ value was calculated at optimum temperature for this species, it is quite low when compared to that of other species such as *A. gossypii* (r_m_ = 0.33 to 27° C, Razmjou *et al* 2006), with which it coexists. In the present paper, demographic parameters were estimated from aphids living on mature leaves, thus they could be underestimations because young leaves might be a better source of nutrients, such as nitrogen. However, Rondón et al. (2005) showed that *C. fragaefolii* infest the whole plant, not just young leaves.

**Figure 3:**
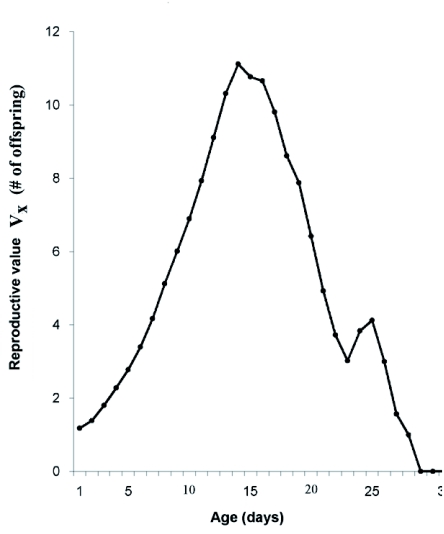
Reproductive value (Vx) of *Chaetosiphon fragaefolii* on strawberry. High quality figures are available online.

The highest reproductive value of mature age (Figure 3) was reached between days 14 and 17; after that offspring production decreased abruptly.

Although the aphids are not the main pests in strawberry crops, *C. fragaefolii* can be a serious problem because it can transmit several virus diseases of strawberry. According to Mellow and Frazier (1970) it can acquire viruses 24 hours after being born. After a latency period of 10 to 20 days, the infected aphid can transmit viruses for up to two weeks. *C. fragaefolii* was not detected during 2007 and this sporadic behavior is particularly dangerous because the aphid may not be detected until it is too late for effective control.

During the course of this study, damage caused by the *strawberry mottle virus* and signs of damage by the *strawberry crinkle virus* were observed (C. Cédola, personal observation). No further information is available about this species in Argentina. Accordingly, these new findings about life traits and mortality factors are useful to design appropriate control strategies for these aphids. This contributes to set the basis for effective pest management.

## References

[bibr01] Begon M, Harper J, Townsed C (1986). *Ecology. Individuals, Populations and Communities*.

[bibr02] Caswell H (1989). *Matrix population models.*.

[bibr03] Coley D, Aide TM, Price PW, Lewinsohn TM, Fernandes GW, Benson WW (1991). A comparison of herbivory and plant defences in temperate and tropical broad-leaved forest.. *Ecology of Plant*—*Animal Interactions: Tropical and Temperate Perspectives*.

[bibr04] Cordo H, Logarzo G, Braun K, Di Dorio O (2004). *Catálogo de Insectos Fitófagos de la Argentina y sus Plantas Asociadas*.

[bibr05] Costamagna A, Landis D (2006). Predators exert a top down control of soybean aphid across a gradient of agricultural management systems.. *Ecological Applications*.

[bibr06] Díaz B, Fereres A (2005). Life table and population parameters of *Nasonovia ribisnigri* (Homoptera: Aphididae) at different constant temperatures.. *Environmental Entomology*.

[bibr07] Kouassi M, Coderre D, Todorova S (2003). Effects of the timing of applications on the incompatibility of three fungicides and one isolate of the entomopathogenic fungus *Beauveria bassiana* (Balsamo) Vuillemin (Deuteromycotina).. *Journal of Applied Entomology*.

[bibr08] Krczal H (1982). Investigations on the biology of the strawberry aphid (*Chaetosiphon fragaefolii*), the most important vector of strawberry viruses in West Germany.. *Acta Horticulturae*.

[bibr09] Krebs C (1999). *Ecological methods*.

[bibr10] Lagnaoui A, Radcliff E (1998). Potato fungicides interfere with entomopathogenic fungi impacting population dynamics of green peach aphid.. *American Journal of Potato Research*.

[bibr11] La Rossa FR, Vasicek AL, Ricci EM (2000). Biology of *Nasonovia ribisnigri* (Homoptera: Aphidoidea) on three varieties of lettuce (*Lactuca sativa*).. *Revista de la Sociedad Entomológica Argentina*.

[bibr12] Mattson WJ, Scriber JM, Slansky F, Rodriguez JG (1987). Nutritional ecology of insect folivores of woody plants: Nitrogen, water, fiber, and mineral considerations. *Nutritional ecology of insects, mites, spiders, and related invertebrates*.

[bibr13] Mellow F, Frazier N (1970). Strawberry mottle.. *Viruses and diseases of small fruits and grapevines*.

[bibr14] Myer S, Gratton C (2006). Influence of potassium fertility on soybean aphids, *Aphis glycines* Matsumura (Hemiptera: Aphididae) populations dynamics at a field and regional scale.. *Environmental Entomology*.

[bibr15] Michels GJ, Behle RW (1988). Reproduction and development of *Diuraphis noxia* (Homoptera: Aphididae) at constant temperatures.. *Journal of Economic Entomology*.

[bibr16] Molina N, Gimenez L, Richieri C (2007). Economía del sector hortícola de Corrientes. Producción frutillera regional y su relación con la oferta nacional y del MERCOSUR.. *Instituto Nacional de Tecnología Agropecuaria- Estación Experimental Bella Vista*.

[bibr17] Moreau G, Eveleigh ES, Lucarotti CJ, Quiring DT (2006). Ecosystem alteration modifies the relative strengths of bottom-up and top-down forces in a herbivore population.. *Journal of Animal Ecology*.

[bibr18] Nowierski RM, Zeng Z, Scharen AL (1995). Age-specific life table modeling of the Russian Wheat Aphid (Homoptera: Aphididae) on barley grown in benzimidazole agar. *Environmental Entomology*.

[bibr19] Nome SF, Yossen V (1980). Identificación de virus de frutilla en Argentina. I. virus del moteado de la frutilla (*Strawberry mottle virus*).. *Revis ta de Investigaciones Agropecuarias*.

[bibr20] Perrenoud S (1990). *Potassium and plant health.*.

[bibr21] Razmjou J, Moharramipour S, Fathipour Y, Mirhoseini S (2006). Demographic parameters of cotton *Aphis gossypii* Glover (Homoptera: Aphididae) on five cotton cultivars.. *Insect Science*.

[bibr22] Rondon S, Cantcliffe D (2004). *Chaetosiphon fragaefolii* (Homoptera: Aphididae): A potential new pest in Florida?. *Florida Entomologist*.

[bibr23] Rondon S, Cantcliffe D, Price J (2005). Population dynamics of the aphid *Aphis gossypii* (Homoptera: Aphididae), on strawberry grown under protected structure.. *Florida Entomologist*.

[bibr24] Slansky F, Rodriguez JG (1987). Nutritional ecology of insects, mites, spiders and related invertebrates..

[bibr25] von Ende C, Scheiner S, Gurevitch J (2001). Repeated-measures analysis. *Design and analysis of ecological experiments*.

[bibr26] Walker M, Jones T (2001). Relative roles of top-down and bottom-up forces in terrestrial tritrophic plant-insect herbivore-natural enemy systems.. *Oikos*.

[bibr27] Whitham T, Maschinski J, Larson K, Paige K, Price P, Lewinsohn T, Fernandez G, Benson W (1991). Plant response to herbivory: the continuum from negative to positive and underlying physiological mechanisms.. *Plant-Animal Interactions. Evolutionary Ecology in Tropical and Temperate Regions*.

